# Case Report: Successful control of ocular surface squamous neoplasia complicated by *Alternaria alternata* infection

**DOI:** 10.3389/fmed.2026.1943460

**Published:** 2026-09-23

**Authors:** Shengnan Liu, Wei Tang, Zhao Gao, Zhongkai Hao, Mingming Liu, Shuang Song, Qiaoling Wang, Jing Wang, Hui Zhang, Hui Dang

**Affiliations:** Department of Ophthalmology, Jinan Second People’s Hospital, Jinan, Shandong, China

**Keywords:** *Alternaria alternata*, corneoscleral collagen cross-linking, cryotherapy, fungal infection, ocular surface squamous neoplasia

## Abstract

**Background:**

Ocular surface squamous neoplasia (OSSN) is relatively common, but the cases complicated by fungal infection are quite rare. This study reviewed a rare case of OSSN complicated by *Alternaria alternata* infection.

**Case presentation:**

A 59-year-old male patient presented with a one-month history of foreign body sensation and photophobia in the left eye. Diagnostic and evaluative procedures, including a series of pathogenic microorganism staining microscopy and culture, *in vivo* confocal microscopy, anterior segment optical coherence tomography, ultrasound biomicroscopy, and histopathological examination, were performed to assess the condition of the cornea, conjunctiva, and sclera. The patient received topical and systemic medication to control infection. Subsequently, he underwent surgical intervention for the left eye consisting of excision of the corneal and conjunctival lesions, cryotherapy, corneoscleral collagen cross-linking, and conjunctival autograft transplantation. Fungal culture of the conjunctival lesion tissue confirmed that the pathogen was *Alternaria alternata* (AA). Histopathological examination revealed ocular surface squamous neoplasia. The diseases were successfully controlled.

**Conclusion:**

To our knowledge, this is the first case report of OSSN with *Alternaria alternata* infection. Microbiological culture remains the gold standard for diagnosing ocular infectious diseases. For this patient, the combined treatment achieved favorable local clinical control. This highlights the importance of combining multiple treatment methods when dealing with such cases.

## Introduction

Ocular surface squamous neoplasia (OSSN) is an umbrella term encompassing ocular surface epithelial intraepithelial neoplasia, carcinoma *in situ*, and invasive squamous cell carcinoma (SCC) (1). Based on morphological features, OSSN can present as gelatinous, papillomatous, leukoplakic, or nodular lesions with superficial vascular growth, or it may present as diffuse lesions with a protracted course and slow growth. OSSN can also masquerade as chronic blepharitis, conjunctivitis, pterygium, necrotizing scleritis, keratitis, or superior limbic keratoconjunctivitis, potentially leading to misdiagnosis and delayed treatment (2). The risk factors for OSSN include prolonged exposure to ultraviolet B (UV B) radiation, aging, human immunodeficiency virus (HIV) infection, human papillomavirus (HPV) infection, vitamin A deficiency, ocular surface damage, and exposure to petroleum chemicals, etc (3). Additionally, chronic ocular surface inflammation may be a potential risk factor for OSSN (4). There have been reports of a rare case of OSSN occurring after chronic fungal keratitis (5).

*Alternaria alternata* (AA) is a type of fungus commonly found in soil and parasitic on plants. It often triggers IgE-mediated allergic diseases (6), and may lead to skin and subcutaneous infections, ocular fungal infections, sinusitis, and onychomycosis. Occupational exposure to soil and garbage, surgical and non-surgical trauma, as well as local or systemic use of glucocorticoids, are predisposing factors for ocular fungal infections (7).

In this study, we report a case of OSSN with AA infection. A series of examinations helped to further diagnose the condition. Through medication and surgery, the condition was successfully controlled.

## Case description

The patient (male, 59 years old, farmer) presented with a one-month history of left-eye foreign body sensation and photophobia without any obvious cause. He had visited a local hospital and was diagnosed with “left eye conjunctivitis”. He was treated with topical application of pranoprofen 0.1% eye drops and tobramycin dexamethasone eye drops for two days, but the effect was not satisfactory. On September 16, 2024, the patient visited our hospital. A sample of the lesion tissue around the conjunctiva was taken for microscopic examination and culture of pathogenic microorganisms, as well as for fungal smear microscopic examination: fungal hyphae were detected. The doctor recommended that the patient receive comprehensive inpatient treatment. However, the patient immediately refused, stating that their financial situation was poor. Therefore, a relatively inexpensive fluconazole 0.5% eye drops (qid) was empirically administered for local eye treatment. After much deliberation, the patient was admitted to the hospital on September 18, 2024, and was diagnosed with “1. Corneal tumor (left eye) 2. Conjunctival tumor (left eye) 3. Suspected fungal conjunctivitis (left eye)”. The patient had a history of atrial fibrillation for six months and has been treated with oral medication under adequate control. He denied having hypertension, diabetes mellitus, systemic immune diseases, or any history of ocular trauma or surgery.

After admission, a series of specialized ophthalmic examinations were conducted. The uncorrected visual acuities (UCVA) of both eyes were 0.00 LogMAR. The patient underwent slit lamp examination of the left eye, as shown in Figure 1A. Under slit lamp examination, no other ocular abnormalities were found in the anterior and posterior segments of both eyes. The intraocular pressure (IOP) was measured using the non-contact tonometer (NCT), with 16 mmHg in the right eye (OD) and 15 mmHg in the left eye (OS). The results of ultrasound biomicroscopy (UBM), anterior segment optical coherence tomography (AS-OCT), and *in vivo* confocal microscopy (IVCM) are shown in Figures 2A–C.

**Figure 1:**
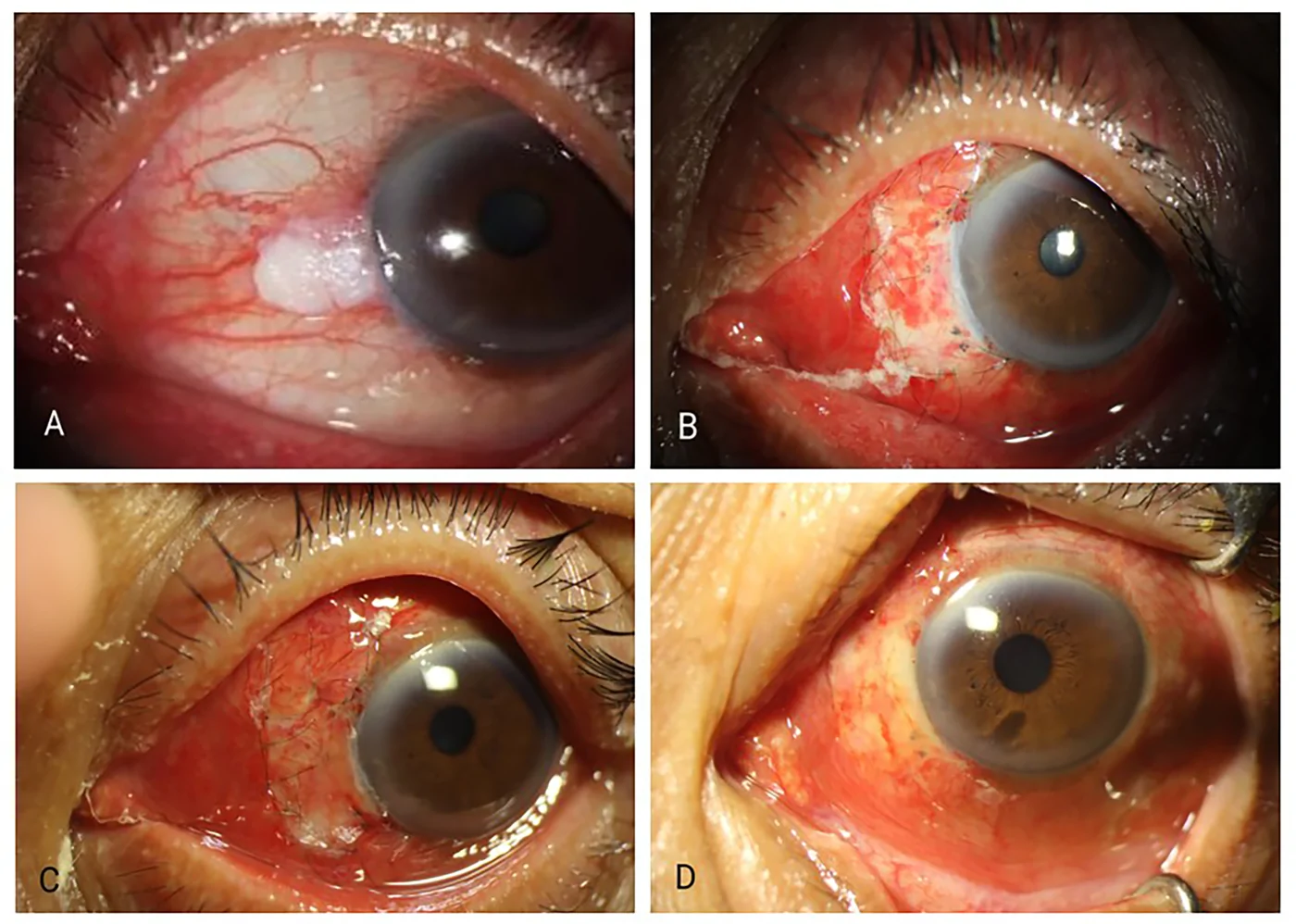
The anterior segment images of the left eye at different treatment stages. **(A)** At initial presentation, there was an irregular hyperplasia of the bulbar conjunctiva at 9 o'clock near the limbus, with white pseudomembrane-like substances visible on the surface, and infiltration at the limbus with unclear boundaries. **(B)** On the first day after surgery, the conjunctiva was congested, the sutures were in place, the wound was well apposed, and there was partial corneal epithelial defect in the surgical area with a smooth surface. **(C)** On the third day after surgery, the conjunctival congestion was reduced, the sutures were in place, the wound was well apposed, and the corneal epithelium was basically repaired. **(D)** One week after surgery after suture removal, there was mild conjunctival congestion, the wound healed well, and the corneal epithelium was repaired.

**Figure 2:**
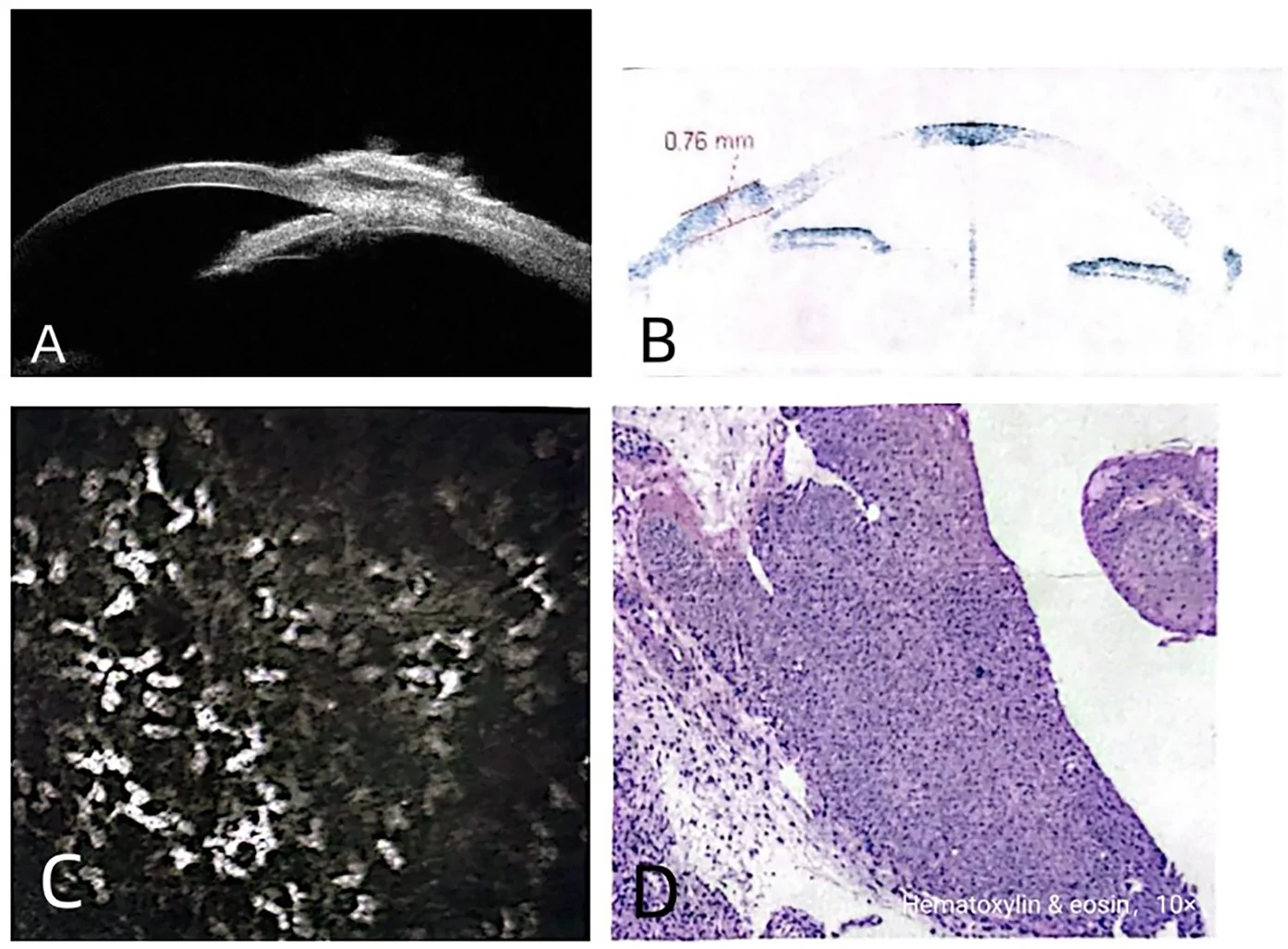
**(A)** UBM image. A raised lesion is visible at approximately 9 o'clock in the conjunctiva and corneal limbus of the left eye, and the echography of the sclera is discontinuous. **(B)** AS-OCT image. A raised lesion is visible at the nasal corneal limbus of the left eye, approximately 0.76 mm high, infiltrating the cornea, with an unclear boundary and uneven internal echo. **(C)** IVCM image. Inflammatory cells are visible in the lesion area,with no definitive fungal hyphae observed. **(D)** Histopathological examination using hematoxylin and eosin (H&E) staining. In the lesion area, part of the squamous epithelium shows high-grade intraepithelial neoplasia, with an intact basement membrane and no stromal invasion. There is mild lymphocytic and plasma cell infiltration in the interstitium. No lesions are found at the surgical margins.

Pending the results of bacterial and fungal culture and bacterial susceptibility testing, the patient received empirical combination antimicrobial therapy comprising: topical levofloxacin 0.3% eye drops (qid) for broad-spectrum antibacterial coverage; pranoprofen 0.1% eye drops (qid) for anti-inflammatory control; and natamycin 5% eye drops (qid), supplemented by oral itraconazole 200 mg (qd) as primary antifungal therapy. On September 20, 2024, the bacterial culture of the conjunctival lesion tissue showed no bacterial growth, and the fungal culture result of the conjunctival lesion tissue was identified as infection by AA (Figures 3A,B). Subsequently, on September 20, 2024, the patient underwent surgical treatment. Under local anesthesia, the neoplastic lesions of the cornea and conjunctiva were excised using a no touch technique with 2 mm margins. During the operation, under the 10x magnification of the surgical microscope, the sclera was observed to be intact and no lesion infiltration was detected. Necrotic tissue within the zone of corneal stromal infiltration was debrided, followed by adjunctive cryotherapy applied to the limbal margin and conjunctival resection borders to mitigate local recurrence risk. The corneoscleral lesion area was immersed in riboflavin 0.1% solution for 10 min. The “continuous” treatment mode was selected, with the irradiance of 30 mW/cm^2^ and the total radiant exposure of 7.2 J/cm^2^. The irradiation probe of the cross-linking device was adjusted to target the corneal and scleral lesion area within an approximate 8 mm diameter, with an irradiation time of 4 min. Under the 10x magnification of the surgical microscope, it was observed that the conjunctival tissue in the superotemporal quadrant far from the lesion showed no obvious abnormalities in terms of morphology and structure. A 6 × 6mm^2^ conjunctival graft was taken from the superotemporal quadrant with the corneal limbus as the base. It was stretched and positioned to cover the conjunctival defect, and then sutured and fixed with 10–0 thread. Erythromycin ophthalmic ointment was instilled into the conjunctival fornix, and the left eye was bandaged with a sterile patch. The excised lesion specimen was submitted for histopathological examination. Postoperatively, recombinant bovine basic fibroblast growth factor ophthalmic gel (21,000IU/5 g, qid) was administered in addition to the continuation of preoperative medical therapy to support corneal epithelial wound healing. The patient exhibited progressive clinical improvement (Figures 1B,C).

**Figure 3:**
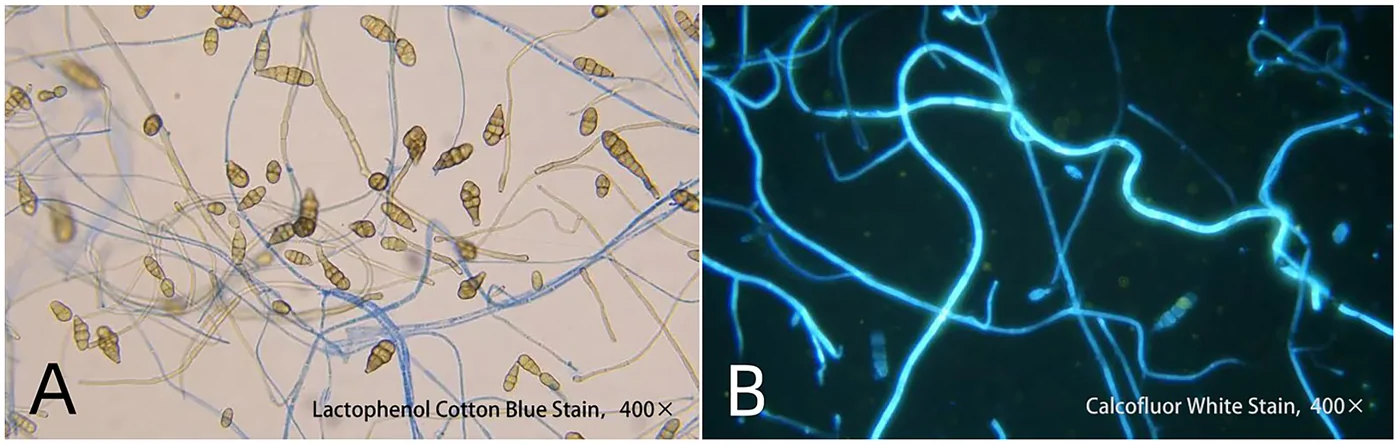
Microscopic morphology of *Alternaria alternata* isolated from fungal culture. **(A,B)** Characteristic multicellular, muriform conidia with transverse and longitudinal septation are evident under Lactophenol Cotton Blue Stain and Calcofluor White Stain.

At the one-week follow-up, the patient's ophthalmic examination showed that the UCVA was 0.00 LogMAR (OS). The slit lamp examination showed that the left eye presented with mild conjunctival congestion, the sutures were intact, the surgical incision had fully regenerated and was well apposed, the corneal epithelium had recovered, the basement membrane structure was intact, and the unaffected stroma of the cornea remained transparent. The IOPs were 15mmHg (OD) and 15mmHg (OS). Sutures were removed (Figure 1D). Pathological results showed: (Cornea, conjunctiva) Some parts of the squamous epithelium presented high-grade intraepithelial neoplasia (Figure 2D). Periodic Acid–Schiff (PAS) staining: (-). Instruct the patient to gradually reduce the dosage of topical levofloxacin 0.3% eye drops and natamycin 5% eye drops to a level where they can be discontinued after 4 weeks post-operation. Apply topical pranoprofen 0.1% eye drops and oral itraconazole 200 mg until they are stopped 2 weeks after the operation. The patient was advised to return for a follow-up visit in two weeks. However, the patient did not show up as scheduled. During the six-month and one-year postoperative follow-ups by phone, the patient reported that he did not show any symptoms suggesting recurrence of the lesion. The patient stated that he had been regularly undergoing follow-up eye examinations at the local hospital, but we were unable to retrieve the original examination records for objective verification. The patient was very satisfied with the treatment and was still advised to visit our hospital regularly for follow-up and observation of the changes in his condition.

## Discussion

In this study, the lesion of OSSN was located at the nasal corneal limbus and conjunctiva, which is consistent with the pathogenesis of OSSN. The epithelial recesses at the limbus of the cornea in the most UV-radiated nasal area are densely distributed, and there are a large number of basal epithelial stem cells. These may be the precursor cells of OSSN (8). Regarding the occurrence of OSSN in the inflammatory ocular surface environment, the available literature is extremely limited. Chronic inflammation caused by microbial infections, autoimmune diseases, and environmental irritants may be predisposing factors for atypical epithelial hyperplasia. The literature has reported that patients with atopic keratoconjunctivitis developed OSSN after treatment with cyclosporine (9). Chronic inflammation may have affected the homeostasis of the limbal basal epithelial stem cells, thereby initiating compensatory hyperplasia. Abnormally proliferating DNA-damaged basal epithelial stem cells develop into atypical hyperplasia (5). The occurrence of OSSN may also damage the defense mechanism of the ocular surface, thereby increasing the risk of opportunistic fungal infections on the ocular surface. AA is increasingly recognized as an opportunistic pathogen in individuals with normal immune function, especially among agricultural workers (7). Given that the patient was engaged in agricultural labor, ocular surface fungal infection was considered the primary differential diagnosis during the initial clinical assessment. At present, the exact causal relationship cannot be determined. However, in the eyes with chronic inflammation, OSSN may be overlooked, and clinicians need to be vigilant about the occurrence of OSSN.

The clinical symptoms and signs of ocular surface inflammation and OSSN lack obvious specificity. Further diagnosis requires the use of laboratory tests and auxiliary detection methods. The precise identification of pathogens is a multi-step process. In clinical practice, microbiological techniques are often used for objective etiological identification, mainly including staining and microscopic examination of pathogenic microorganisms, culture, and antibacterial susceptibility testing. In this study, routine procedures such as pathogen staining microscopy, bacterial and fungal culture, and bacterial susceptibility testing were carried out. The staining microscopy of pathogenic microorganisms around the conjunctival lesion showed the presence of fungal hyphae, and the fungal culture indicated AA infection. The above microbiological evidences indicate the presence of fungal infection. Fungal susceptibility testing was not undertaken, and it is still recommended that clinicians perform this test to better guide clinical medication. The results of fungal susceptibility testing may provide more accurate guidance for clinical treatment. Literatures showed that microbial culture took a relatively long time and had a high false negative rate, with more than 50% of cases unable to identify pathogenic microorganisms through culture. The results of microbial detection are not always reliable (10). Therefore, additional auxiliary detection methods are still needed to further improve the diagnosis. IVCM has been used for the early diagnosis of fungal keratitis. However, this equipment highly depends on the operator's skills. Misdiagnosis may occur due to artifacts (11). The IVCM test results of this patient showed no obvious fungal hyphae, and inflammatory cells were observed in the affected area. However, this still does not rule out the possibility of fungal keratitis infection. AS-OCT can be used to assess the depth of infiltration before treatment and monitor the disease progression, but it cannot identify the pathogen (10). When deep ulcers or large infiltration foci affect the assessment of deep ocular tissues, UBM can be used in combination (12). In this study, UBM examination revealed discontinuous scleral echography. However, during the intraoperative microscopic observation, it was observed that the scleral tissue was intact and no obvious lesions were found to infiltrate into the sclera. Subsequent histopathological examination further confirmed that the conjunctival basal membrane was intact and there was no interstitial infiltration. Therefore, the discontinuous scleral echography shown on UBM might be caused by local inflammatory changes induced by OSSN lesions, rather than the actual invasion of the tumor into the sclera. Additionally, AS-OCT can detect OSSN and monitor the regression of OSSN during drug treatment. The diagnosis of OSSN currently mainly relies on clinical suspicion, and is confirmed through methods such as AS-OCT, cytology, and histopathological examination. Histopathological examination is the gold standard for the diagnosis of OSSN (13).

The infection caused by AA responds well to conventional antifungal drugs (7). The antifungal drugs of the polyene and azole classes have broad antifungal effects against various filamentous fungi and yeasts, and thus play an important role in the antifungal treatment of ophthalmology (14). Fluconazole is regarded as an anti-fungal drug with high cost-effectiveness and good safety. It has lower toxicity compared to other azole antifungal drugs. It shows efficacy in the treatment of fungal keratitis (14). In this study, the attending physician took the patient's financial situation into account and selected fluconazole for local treatment of the suspected filamentous fungal infection of the ocular surface. If the patient's financial condition permits, perhaps it would be a better option to use natamycin or voriconazole. Natamycin is a first-line medication for treating fungal keratitis. Due to its limited corneal/stromal penetration, it is only suitable for the treatment of superficial infections (15). Voriconazole is the preferred azole drug for treating fungal eye diseases (14). Typically, itraconazole is used as a systemic adjuvant medication for local or intraocular antifungal treatment (15). In this study, both local antifungal treatment and oral itraconazole were administered, with the expectation that the combination of systemic and local treatments would lead to a better therapeutic response. In this study, while using traditional antifungal drugs, pranoprofen was also applied topically for adjunctive anti-inflammatory treatment.

The recurrence rate of the tumor usually increases with the severity of the OSSN pathological grade and also depends on the completeness of the initial resection. Given the difficulty in accurately determining tumor boundaries and depth, current treatment options for conjunctival epithelial malignancies include tumor resection with or without cryotherapy, local interferon-α2b (IFN), mitomycin-C (MMC), and 5-fluorouracil (5-FU) chemotherapy, as well as radiotherapy. Chemotherapy is often used for patients with residual conjunctival lesions. Radiotherapy can be used for advanced squamous cell carcinoma with intraocular or orbital spread, or when surgery cannot achieve a complete cure. During the operation, local cryotherapy was carried out at the surgical margins. Cryotherapy is currently applied in the treatment of various tumors affecting the eye and its accessory organs. The tumor cells are destroyed through the cryogenic effect, followed by the occlusion of microcirculation, leading to ischemic infarction in both normal and neoplastic tissues (16). In this study, corneoscleral collagen cross-linking was used to control fungal infections and increase the biomechanical strength of the cornea and sclera. Studies have shown that the combination of corneoscleral collagen cross-linking surgery with antifungal drugs can accelerate ulcer healing and reduce medication frequency. However, this therapy may not achieve the expected results in deep stromal infections and is only applicable to superficial infections that do not involve the deeper layers below 300 micrometers (17).

For larger conjunctival tissue defects, ocular surface reconstruction is of great significance in achieving good functional and aesthetic results. In recent years, conjunctival autograft transplantation and amniotic membrane transplantation (AMT) have been widely used in the reconstruction of the ocular surface. AMT is regarded as an effective option for the reconstruction of large-area ocular surface defects after tumor resection. In the existing literature, conjunctival autograft transplantation is more commonly used in the treatment of pterygium (18), as well as in the conjunctival–limbal autograft transplantation for extensive ocular surface tumors to prevent the occurrence of postoperative limbal stem cell deficiency (19). Simple conjunctival autograft transplantation is rarely used in OSSN. In this study, conjunctival autograft transplantation was performed to reconstruct the ocular surface at the site of conjunctival defect. We believe that conjunctival autograft transplantation can also be a viable option in the ocular surface reconstruction of OSSN patients. Compared to the significant drawbacks of AMT (20), conjunctival autograft transplantation has its own clinical advantages, such as being less prone to degradation, having long-lasting activity, being easier to observe, and facilitating rapid epithelialization. During the operation, we used a non-contact technique to expand the resection of the tumorous lesions in the cornea and conjunctiva. At the same time, we performed auxiliary cryotherapy at the resection margins of the corneal limbus and conjunctiva, and carried out corneoscleral collagen cross-linking treatment. We attempted to completely remove the lesions, and the final pathological results also confirmed the completeness of our resection. If surgical margins were assessed by intraoperative frozen-section pathological examination, the surgical outcome may be more reliable. During the operation, it is necessary to thoroughly assess the conjunctival graft and rule out any visible tumors. However, there is still a risk of tumor implantation due to the presence of undetected small lesions.

Our study presented an exceptionally rare case of OSSN complicated by AA infection, which contributed valuable insights and clinical experience regarding this condition. The patient has been followed up for one year. He reports that the condition is stable, but further follow-up is necessary to monitor for possible recurrence. This case report also indicates that it is very important to protect the eyes in work and life, and any eye discomfort should be treated timely. When anti-inflammatory treatment is ineffective, doctors should be vigilant about the occurrence of OSSN.

## Data Availability

The original contributions presented in the study are included in the article/Supplementary Material, further inquiries can be directed to the corresponding author.

## References

[1] Hollhumer R Michelow P Libhaber E Williams S. Management and outcomes of ocular surface squamous neoplasia at a tertiary hospital, South Africa. Eye (Lond). (2025) 39(14):2713–9. 10.1038/s41433-025-03926-8 40715431 PMC12446432

[2] Theotoka D Wall S Galor A Sripawadkul W Khzam RA Tang V. The use of high resolution optical coherence tomography (hr-oct) in the diagnosis of ocular surface masqueraders. Ocul Surf. (2022) 24:74–82. 10.1016/j.jtos.2022.02.003 35231640 PMC9058205

[3] Tsatsos M Delimitrou C Tsinopoulos I Ziakas N. Update in the diagnosis and management of ocular surface squamous neoplasia (ossn). J Clin Med. (2025) 14(5):1699. 10.3390/jcm14051699 40095695 PMC11900158

[4] De Arrigunaga S Wall S Theotoka D Friehmann A Camacho M Dubovy S. Chronic inflammation as a proposed risk factor for ocular surface squamous neoplasia. Ocul Surf. (2024) 33:1–10. 10.1016/j.jtos.2024.02.006 38432640 PMC13409058

[5] Singh S Mittal R Narang P Mittal V. Ocular surface squamous neoplasia in a setting of fungal keratitis: a rare co-occurrence. Int Ophthalmol. (2019) 39(3):717–20. 10.1007/s10792-018-0864-8 29423784

[6] Bartra J Belmonte J Torres-Rodriguez JM Cistero-Bahima A. Sensitization to alternaria in patients with respiratory allergy. Front Biosci (Landmark Ed). (2009) 14(9):3372–9. 10.2741/3459 19273281

[7] Pastor FJ Guarro J. Alternaria infections: laboratory diagnosis and relevant clinical features. Clin Microbiol Infect. (2008) 14(8):734–46. 10.1111/j.1469-0691.2008.02024.x 18727797

[8] Novelli A Romero-Kusabara IL Frazao MAM. Update in ocular surface squamous neoplasia. Rev Assoc Med Bras (1992). (2024) 70(suppl 1):e2024S124. 10.1590/1806-9282.2024S124 38865544 PMC11164286

[9] Flynn TH Manzouri B Tuft SJ. Ocular surface squamous neoplasia in an immunosuppressed patient with atopic keratoconjunctivitis. Int Ophthalmol. (2012) 32(5):471–3. 10.1007/s10792-012-9577-6 22581322

[10] Gheorghe AG Arghirescu AM Marinescu MC Onofrei AG Pop DM Voinea LM. *In Vivo* confocal microscopy and anterior segment optical coherence tomography in optimizing diagnosis and therapeutic management in fungal keratitis: case reports and literature review. J Clin Med. (2025) 14(22):8066. 10.3390/jcm14228066 41303103 PMC12653982

[11] Takezawa Y Shiraishi A Noda E Hara Y Yamaguchi M Uno T. Effectiveness of *in vivo* confocal microscopy in detecting filamentous fungi during clinical course of fungal keratitis. Cornea. (2010) 29(12):1346–52. 10.1097/ICO.0b013e3181cd3c84 20847667

[12] Fogel Levin MM Moroz I Birger Y Hostovsky A Fried S Fabian ID. Ultrasound biomicroscopy and anterior segment oct in ocular surface squamous neoplasia: complementary roles, case series, and targeted literature review. Front Ophthalmol (Lausanne). (2026) 6:1794093. 10.3389/fopht.2026.1794093 41908639 PMC13017243

[13] Hӧllhumer R Williams S Michelow P. Ocular surface squamous neoplasia: management and outcomes. Eye (Lond). (2021) 35(6):1562–73. 10.1038/s41433-021-01422-3 PMC8169825 33564137

[14] Reginatto P Agostinetto GJ Fuentefria RDN Marinho DR Pizzol MD Fuentefria AM. Eye fungal infections: a Mini review. Arch Microbiol. (2023) 205(6):236. 10.1007/s00203-023-03536-6 37183227 PMC10183313

[15] Patil A Majumdar S. Echinocandins in ocular therapeutics. J Ocul Pharmacol Ther. (2017) 33(5):340–52. 10.1089/jop.2016.0186 28437176

[16] Herta J Matula C Wadiura L Millesi M. The value of cryoablation in orbital surgery. Wien Klin Wochenschr. (2025) 137(1-2):16–20. 10.1007/s00508-024-02340-6 38483600 PMC11739241

[17] Barac IR Artamonov A-R Baltă G Dinu V Mehedințu C Bobircă A. Photoactivated chromophore corneal collagen cross-linking for infectious keratitis (pack-cxl)-a comprehensive review of diagnostic and prognostic factors involved in therapeutic indications and contraindications. J Pers Med. (2022) 12(11):1907. 10.3390/jpm12111907 36422083 PMC9698237

[18] Peng W Wang C Gong X Zheng Z Yu K Zhou T. A randomized controlled trial comparing limbal-conjunctival autograft with conjunctival autograft in recurrent pterygium surgery with intraoperative application of 0.02% mitomycin C. Int Ophthalmol. (2026) 46(1):256. 10.1007/s10792-026-04121-8 42257741

[19] Doganay S Er H Tasar A Gurses I. Surgical excision, cryotherapy, autolimbal transplantation and mitomycin-C in treatment of conjunctival-corneal intraepithelial neoplasia. Int Ophthalmol. (2005) 26(1-2):53–7. 10.1007/s10792-006-0006-6 16779567

[20] Korkmaz I Gurdal M Arici M Barut Selver O. Beyond transplants: current and future therapeutic potential of amniotic membrane extract (ame) in ophthalmology. Regen Med. (2025) 20(2-3):97–109. 10.1080/17460751.2025.2472578 40028702 PMC11951707

